# Bardoxolone methyl inhibits the infection of *rabies virus* via Nrf2 pathway activation in vitro

**DOI:** 10.1186/s12985-023-02213-w

**Published:** 2023-11-10

**Authors:** Ying Lin Chi, Yuan Xie, Shu Qing Liu, Wu Yang Zhu

**Affiliations:** Key Laboratory of Medical Virology, Ministry of Health, National Institute for Viral Disease Control and Prevention, NHC Key Laboratory of Biosafety, Chinese Center for Disease Control and Prevention, No.155 Changbai Road, Changping District, Beijing, 102206 People’s Republic of China

**Keywords:** Rabies virus, Bardoxolone methyl, Nuclear factor erythroid-derived 2-like 2, Neuro2a cells

## Abstract

**Background:**

Rabies is a widespread, fatal, infectious disease. Several antivirals against *rabies virus* (RABV) infection have been reported, but no approved, RABV-specific antiviral drugs that inhibit RABV infection in the clinic after symptom onset are available. Therefore, more effective drugs to reduce rabies fatalities are urgently needed. Bardoxolone methyl (CDDO-Me), an FDA-approved compound that has long been known as an antioxidant inflammatory modulator and one of the most potent nuclear factor erythroid-derived 2-like 2 (Nrf2) activators, protects myelin, axons, and CNS neurons by Nrf2 activation. Therefore, we investigated the potency of its anti-RABV activity in vitro.

**Methods:**

The mouse neuroblastoma cell line Neuro2a (N2a) and three RABV strains of different virulence were used; the cytotoxicity and anti-RABV activity of CDDO-Me in N2a cells were evaluated by CCK-8 assay and direct fluorescent antibody (DFA) assay. Pathway activation in N2a cells infected with the RABV strains SC16, CVS-11 or CTN upon CDDO-Me treatment was evaluated by western blotting (WB) and DFA assay.

**Results:**

CDDO-Me significantly inhibited infection of the three RABV strains of differing virulence (SC16, CVS-11 and CTN) in N2a cells. We also examined whether CDDO-Me activates the Nrf2-associated pathway upon infection with RABV strains of differing virulence. Nrf2, phosphorylated sequestosome (SQSTM1), SQSTM1, hemoglobin oxygenase (HO-1) and NAD(P)H dehydrogenase quinone 1 (NQO1) expression in N2a cells increased to varying degrees with CDDO-Me treatment, accompanied by Kelch-like ECH-associated protein 1 (Keap1) dissociation, upon infection with SC16, CVS-11 or CTN. The activation of SQSTM1 phosphorylation was significantly associated with the degradation of Keap-1 in CDDO-Me-treated N2a cells upon RABV infection. Furthermore, N2a cells pretreated with the Nrf2-specific inhibitor ATRA showed a significant decrease in HO-1 and NQO1 expression and a decrease in the anti-RABV efficacy of CDDO-Me. These inhibitory effects were observed upon infection with three RABV strains of differing virulence.

**Conclusion:**

CDDO-Me inhibited RABV infection via Nrf2 activation, promoting a cytoprotective defense response in N2a cells. Our study provides a therapeutic strategy for RABV inhibition and neuroprotection during viral infection.

## Introduction

Rabies is a widespread infectious disease that continues to confer a serious public health burden, causing more than 55,000 human deaths worldwide each year [1]. All mammals are believed to be susceptible to rabies virus (RABV) infection, which has a case-fatality rate of almost 100% in nonvaccinated individuals [2, 3]. RABV, a member of the Lyssavirus genus of the Rhabdoviridae family and the causative agent of rabies, is highly neuroinvasive and usually causes lethal central nervous system (CNS) disease [4, 5]. Pre- or postexposure vaccination remains the only way to prevent rabies.

Known antiviral agents, such as amantadine and interferon alpha, have failed to demonstrate therapeutic efficacy in a mouse model and clinical cases of RABV [6]. The compound 1,2,3,4,6-penta-O-galloyl-β-D-glucose (PGG) demonstrated potent anti-RABV activity in cell cultures and RABV-infected mice [7]. Ribavirin, a broad-spectrum guanine nucleoside analog, inhibited RABV replication in vitro but failed to demonstrate protective efficacy in human clinical cases [5]. The ribavirin-related compounds EICAR and EICNR have shown greater anti-RABV activity than ribavirin, which inhibited RABV in vitro [6]. TMR-001 was found to inhibit RABV in vitro but did not prevent RABV transit from the periphery to the CNS in a Syrian hamster model [8]. Favipiravir (T-705), another nucleoside analog, inhibited RABV in vitro but showed limited application value as a single therapeutic agent for rabies in a mouse model [9, 10], as T-705 suppressed RABV replication in the periphery, but a large dose was required for it to be effective in the CNS [9]. Therefore, more compounds for the treatment of rabies, especially in patients with symptomatic rabies, need to be identified.

Oleanolic acid, a naturally occurring triterpenoid, has been used in traditional medicine for centuries and has shown antioxidant, antibacterial, antifungal, anticancer, and anti-inflammatory activities [11]. A series of novel oleanolic acid derivatives have been synthesized to improve its pharmacological efficacy; these derivatives include 2-cyano-3,12-dioxooleana-1,9(11)-dien-28-oic acid (CDDO) and bardoxolone methyl (CDDO-Me) [12]. CDDO-Me has long been known for its anti-inflammatory and anticancer activity and exhibits dual effects, as it has anti-inflammatory effects at lower concentrations and is extremely cytotoxic against various cancer cell lines at higher concentrations [13]. CDDO-Me, an antioxidant inflammation modulator and one of the most potent nuclear factor erythroid-derived 2-like 2 (Nfe2l2; Nrf2) activators [14, 15], plays a crucial role in the protection of myelin, axons, and neurons in the CNS by Nrf2 activation [16, 17]. Nrf2 is expressed in all cell types and regulates the expression of over 200 genes; thus, Nrf2 is the master regulator of the cellular antioxidant response [18]. The covalent cysteine modification of Kelch-like ECH-associated protein 1 (Keap1) affects the conformation of Keap1, thus interfering with its binding to Nrf2. Then, newly synthesized Nrf2 accumulates and translocates to the nucleus, where it activates its target genes, including heme oxygenase 1 (HO-1) and NAD(P)H:quinone oxidoreductase 1 (NQO1) [19, 20]. Studies have also found that the regulatory role of Nrf2 involves sequestosome 1 (SQSTM1) or the autophagy adapter protein p62, which plays a role in controlling Keap1 turnover [21, 22]. SQSTM1 phosphorylated at Ser349 can compete with Nrf2 for Keap1 binding [21–23]. CDDO-Me is more potent than CDDO in terms of its anticancer and cancer-preventive activities and ability to activate the Keap1/Nrf2/ARE pathway, which is involved in cytoprotection in the presence of excessive electrophiles or oxidative stress [14, 24]. The effects of CDDO-Me on inflammatory disorders, neurodegenerative diseases, cancers, diabetes and many other diseases have been examined. A study showed that CDDO-Me may exert its beneficial effect by increasing the expression of Nrf2 and HO-1 to protect against ischemic acute kidney injury (AKI) [25]. Moreover, an extended study demonstrated that CDDO-Me could reduce intracranial hemorrhage volume after cerebral ischemia/reperfusion injury and protect the cellular components of the blood–brain barrier (BBB) [26]. Limited studies reported that CDDO-Me induced growth arrest and apoptosis in several neuroblastoma cell lines [27, 28].

Nrf2 has been suggested to be a pivotal host factor involved in protection against virus-induced injury that is essential to mediate the antioxidant system during oxidative injury and inflammation. Phosphorylated SQSTM1 binds with high affinity to Keap1, which inhibits Keap1-driven ubiquitination of Nrf2 and subsequently results in stabilization of Nrf2. Then, Nrf2 translocates into the nucleus to induce the transcription of numerous Nrf2-dependent antioxidant genes, including NQO1, HO-1, glutathione peroxidase (pHGPx), the regulatory and catalytic subunits of glutamate-cysteine ligase (GCLM and GCLC) and glutathione S-transferase A1 (GSTA1) [23, 29–33]. A study provided compelling evidence of the important regulatory role of Nrf2 in the host defense mechanism against respiratory syncytial virus (RSV) disease in a murine model [34]. Yageta Y et al. reported that Nrf2 is a pivotal host factor involved in protection against the development of chronic obstructive pulmonary disease (COPD) exacerbations after influenza virus (FluV) infection and injury under oxidative conditions [35]. The expression levels of NQO1, HO-1, GCLC and GCLM were significantly lower in Nrf2-deficient mice after exposure to FluV and cigarette smoke. Tetsuya Saito et al. found that SQSTM1 phosphorylated at Ser349 accumulated in tumor regions positive for hepatitis C virus (HCV) [29]. Carvajal-Yepes et al. identified a novel mechanism of Nrf2 regulation and suggested that HCV inhibits the induction of the Nrf2/ARE-regulated genes NQO1, GI-GPx, or GCLM, which promote HCV-associated pathogenesis [36]. Zhu, Z. and Lehmann, E. et al. also demonstrated that strong overexpression of HO-1, which resulted in increased biliverdin levels, suppressed HCV replication. The HO-1 product biliverdin triggers the antiviral interferon response and thereby reduces HCV replication [31, 32]. Marburg virus (MARV) hijacks the Nrf2-antioxidant defense system by targeting the Nrf2-negative regulator keap1. Activation of the Nrf2-dependent pathway driven by the MARV protein VP24 could alleviate Keap1-mediated inhibition of the expression of both NQO1 and HO1 in a dose- and time-dependent manner in vitro and likely contributed to the dysregulation of host antiviral inflammatory responses to ensure the survival of MARV-infected cells [37]. Hongsheng Z et al. showed that Nrf2 could negatively regulate Tat-induced HIV-1 LTR transactivation, which might be an important mechanism leading to its anti-HIV-1 activity [38]. In this study, we investigated the antiviral efficacy of CDDO-Me in the Neuro2a (N2a) cell line, which is the principal target cell of RABV. We found that CDDO-Me suppressed viral growth via Nrf2-dependent signaling and inhibited RABV infection in N2a cells. For comparison, we also included ribavirin and T-705, two antiviral compounds previously that were reported to inhibit RABV, in this study [5, 9]. Our results provide strong evidence that CDDO-Me effectively inhibited RABV infection in vitro.

## Materials and methods

### Cell culture and reagents

The mouse neuroblastoma cell line N2a (C1300 clone) was purchased from ATCC (CCL-131) and maintained in ATCC-formulated Dulbecco's modified Eagle medium (DMEM, ATCC) with 10% fetal bovine serum (FBS, Gibco) at 37 °C in a humidified 5% CO_2_ atmosphere. CDDO (MedChemExpress, USA), CDDO-Me (MedChemExpress, USA), ribavirin (MedChemExpress, USA), T-705 (MedChemExpress, USA) and retinoic acid (ATRA, MedChemExpress, USA) were all purchased from MedChemExpress. Each stock was dissolved in DMSO to prepare stock solutions at 10 mM. The stock solutions were freshly diluted with culture medium containing a final concentration of 0.05% DMSO (v/v). The control cells were treated with only 0.05% DMSO.

### Cell viability assay

The Cell Counting Kit-8 (CCK-8; Dojindo, Mashikimachi, Japan) assay was used to analyze cell viability in the absence or presence of CDDO-Me or CDDO in the culture medium according to the manufacturer’s instructions. Briefly, after they reached 70–80% confluence, approximately 1 × 10^5^ N2a cells were seeded, and the culture medium was replaced with 100 μL of a freshly prepared CDDO-Me or CDDO suspension that had been diluted to the appropriate concentration (0.02 μM, 0.05 μM, 0.1 μM, 0.2 μM, 0.5 μM, or 1 μM) with culture medium. Then, the N2a cells were incubated for 24 h. N2a cells treated with DMSO served as a control in the experiment. A commercially available CCK-8 kit was used to evaluate the cytotoxic effects of CDDO-Me and CDDO. The absorbance values of the supernatants at 450 nm were measured with a FLUOstar Omega instrument (ORFLO Technologies, USA). All experiments were performed in triplicate.

### Virus strains

Three RABV strains were used in this study, namely, SC16, CVS-11 and CTN. These strains were chosen for this study because their isolation background, genetic characteristics, and genomic information are known and complete. The RABV strains were maintained in our laboratory. SC16 is a street strain that was isolated from a dog with rabies in Sichuan Province in 2006 [39, 40]. The standard challenge virus (CVS-11) was provided by the National Institutes for Food and Drug Control, China [40]. The parental virus of the CTN strain was isolated from a rabid patient and attenuated by passage in mouse and human diploid cells. The amino acid residue at position 333 of the glycoprotein, which is related to pathogenesis, was mutated from arginine (R) to glutamine (Q). This CTN strain has been approved as a vaccine strain by the World Health Organization (WHO) since 1983 [41, 42]. The strains are ordered as follows in terms of relative neuroinvasiveness and neurovirulence: SC16 > CVS-11 > CTN.

### Virus titration

N2a cells were plated in 96-well culture plates. The cells were incubated with the different strains at an MOI of 10 suspended in test medium for 1 h at 37 °C, washed, and incubated with culture medium. After 72 h of incubation, the viral titer in the N2a cells was determined using a focus assay as previously described [6]. Viral foci were detected using a FITC-conjugated anti-RABV N antibody (Fujirebio Diagnostics, Inc., Malvern, PA, USA). The foci were counted to determine the viral titer (FFU/ml).

### Antiviral assay

N2a cells were incubated until they reached 70–80% confluence and then infected with RABV (SC16, CVS-11 or CTN) at an MOI of 10 in the presence of CDDO-Me diluted to the appropriate concentration (0.02 μM, 0.05 μM, 0.1 μM, 0.2 μM or 0.5 μM) in the culture medium in triplicate. Cells infected with the virus and treated with DMSO served as a control. At 24 h postinfection (hpi), the cells were fixed with 10% formaldehyde and stained with FITC-conjugated anti-RABV N antibody as described previously [43]. The fluorescence signals were detected using an Olympus IX70 inverted fluorescence microscope (Olympus, Tokyo, Japan). The stored supernatant was titrated as described previously [8, 44]. The 50% cytotoxic concentration (CC_50_), which is the concentration of a compound at which the compound reduces cell viability by 50%, was calculated using nonlinear regression analysis of the dose–response curves. The 50% inhibitory concentration (IC_50_) was calculated by regression analysis of the dose–response curves. All experiments were performed with at least three independent replicates. The selectivity index (SI) was calculated by dividing the CC_50_ by the IC_50_ for each compound and virus tested as described previously [45].

### Western blotting

N2a cells were incubated and infected with SC16, CVS-11 or CTN at an MOI of 10 for the indicated duration. The cells in each group were lysed and purified using the M-PER mammalian protein extraction kit (Pierce; Rockford, IL, USA). All samples were supplemented with a proteinase inhibitor (Pierce; Rockford, IL, USA) and a phosphatase inhibitor (Roche; Indianapolis, IN, USA). Extracted proteins were separated by SDS‒PAGE and transferred to polyvinylidene difluoride membranes (Millipore Corp., Bedford, MA, USA). After blocking with 5% nonfat milk in TBST (20 mM Tris − HCl, 15 mM NaCl, 0.05% [v/v] Tween-20, pH 7.4), the membranes were incubated with primary antibodies overnight at 4 °C. Antibodies against the following were used: Nrf2, phospho-SQSTM1, SQSTM1, Keap-1, HO-1, NQO1, and β-actin (all from Cell Signaling Technology; Beverly, MA, USA). The membranes were washed with TBS and then incubated with the appropriate HRP-conjugated goat anti-rabbit IgG secondary antibody (Pierce; Rockford, IL, USA, 1:2000) for 1 h at 25 °C. Protein bands were visualized with SuperSignal West Dura Extended Duration Substrate (Pierce; Rockford, IL, USA). The chemiluminescence signals were detected using a Bio-Rad ChemiDoc™ XRS system, and the blots were analyzed using Image Lab 3.0 (Bio-Rad).

### Statistical analysis

Data were analyzed with SPSS 17.0 and GraphPad Prism software (version 9.0). One-way analysis of variance (ANOVA) was used to analyze the results, and the results were considered to be statistically significant if P < 0.05.

## Results

### Cytotoxicity of CDDO-Me in vitro

**Fig. 1:**
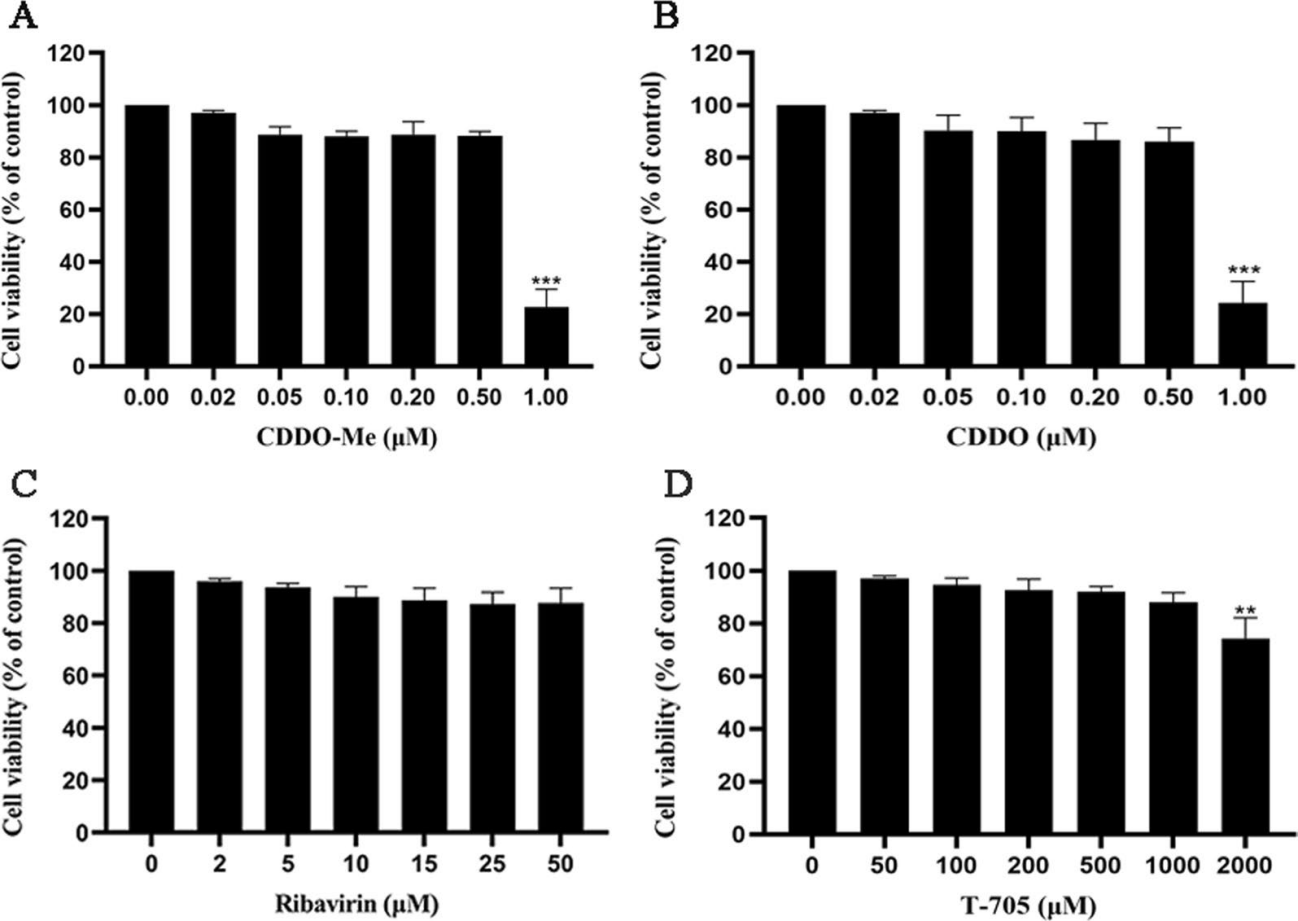
CDDO-Me, CDDO, ribavirin and T705 decreased the viability of N2a cells. N2a cells were incubated with CDDO-Me, CDDO, ribavirin or T705 at varying concentrations for 24 h. Cell viability was examined by CCK-8 assay to evaluate the cytotoxic effects of CDDO-Me **A**, CDDO **B**, ribavirin **C** and T705 **D** according to the manufacturer’s instructions. N2a cells treated with DMSO served as a control in the experiment. One-way ANOVA followed by Dunnett’s multiple comparison test was applied (*p < 0.05, **p < 0.01, ***p < 0.001)

The cytotoxicity of CDDO-Me was examined in N2a cells. Due to the known toxicity of CDDO-Me in N2a cells, CDDO (a CDDO-Me analog) was also evaluated. The cytotoxicity assay showed that the CC_50_ values of CDDO-Me and CDDO were 0.78 ± 0.275 μM and 0.71 ± 0.157 μM, respectively, with no apparent cytotoxicity observed at working concentrations of up to 0.5 μΜ (Fig. 1 A, B; Table 1). The anti-RABV activities of ribavirin and T-705 have been previously demonstrated [6, 14, 46], so ribavirin and T-705 were used as reference compounds in the current study. The cytotoxicity of ribavirin and T-705 was examined in N2a cells, and no cytotoxic effects were observed at the highest concentrations tested (ribavirin at 50 μM and T-705 at 1000 μM) (Fig. 1 C, D; Table 1).

**Table 1:** The cytotoxicity, anti-RABV effect, and selectivity indices of the tested compounds

Compounds	CC_50_(μM)	IC_50_(μM)	SI(CC_50_/IC_50_)
CDDO-Me	0.71 (0.553–0.867)	0.0445 (0.0414–0.0476)	9.59
CDDO	0.78 (0.505–1.055)	0.0620 (0.0467–0.0773)	8.13
Ribavirin	> 50	29.36 (27.21–31.51)	> 1.70
T-705	> 2000	367.50 (360.64–374.36)	> 5.44

CC_50_: 50% cytotoxic concentration; IC_50_: 50% inhibitory concentration; SI: selectivity index; CDDO-Me: bardoxolone methyl; CDDO: bardoxolone; T-705: favipiravir

### Antiviral activity of CDDO-Me in vitro

The IC_50_ values of CDDO-Me, CDDO, ribavirin and T-705 as determined by antiviral assay (Table 1) were 0.0445 ± 0.0031 μΜ, 0.0620 ± 0.0153 μΜ, 29.36 ± 2.15 μΜ and 367.50 ± 6.86 μΜ, respectively. These results showed that CDDO-Me and CDDO had significant anti-RABV activity in N2a cells. Because CDDO-Me was reported to be more potent than CDDO as a cytoprotective agent [14, 24], CDDO-Me was used in subsequent experiments.

**Fig. 2:**
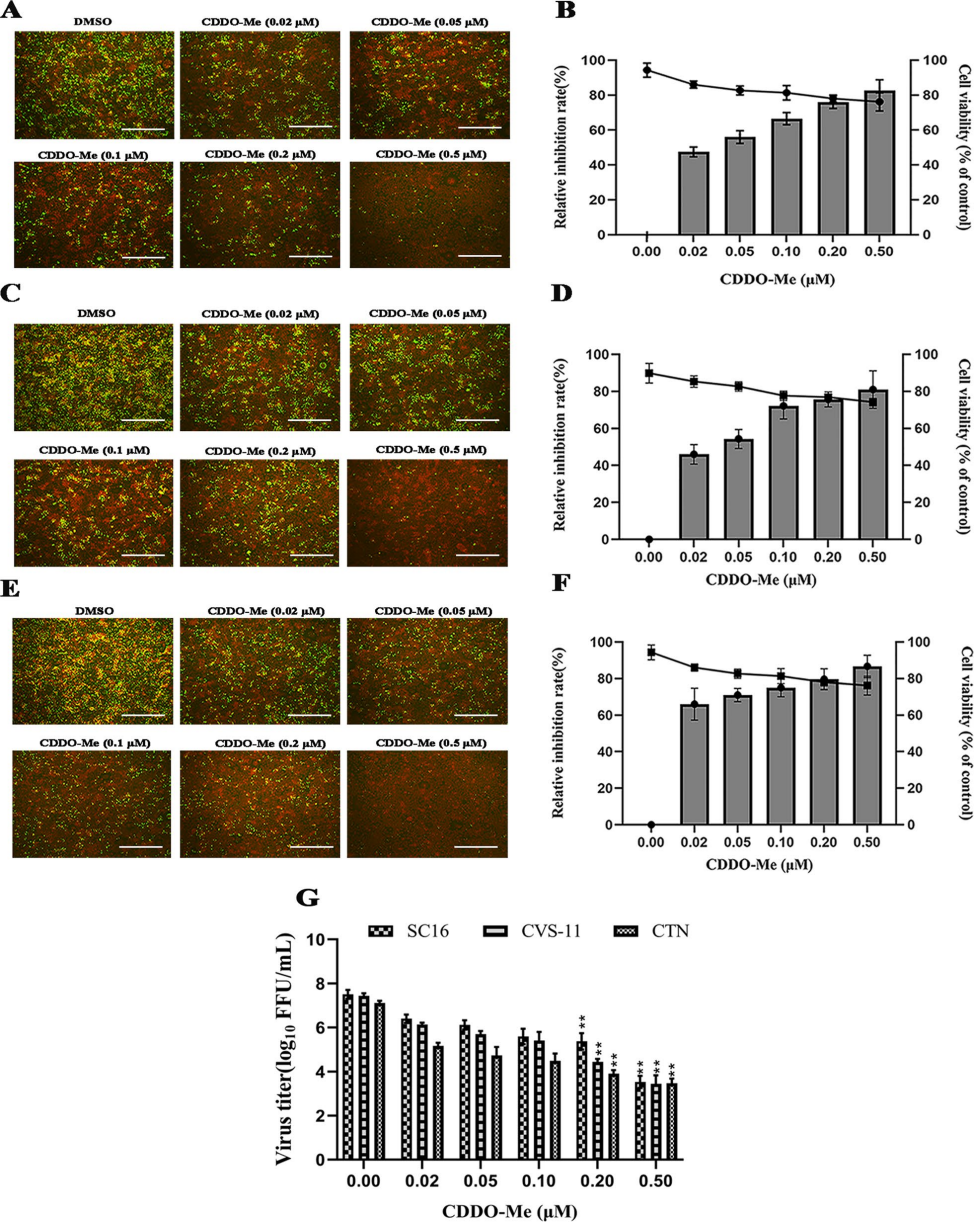
Cytotoxic and inhibitory effects of CDDO-Me in N2a cells infected with different RABV stains. N2a cells were infected with SC16 **A**, CVS-11 **C** or CTN **E** at an MOI of 10 and treated with CDDO-Me diluted in the culture medium to the appropriate concentration (0.02 μM, 0.05 μM, 0.1 μM, 0.2 μM or 0.5 μM) for 24 h in triplicate. Virus-infected cells that were treated with DMSO served as a control. The culture supernatants were harvested, and the viral particles were detected by direct fluorescent antibody (DFA) assay; apple-green fluorescence represents fluorescein isothiocyanate (FITC)-labeled RABV, and the scale bar represents 100 μm. **B**, **D**, **F** The anti-RABV effects of CDDO-Me are presented on the right as the relative inhibition rate, which was calculated by counting clusters of fluorescent foci using DFA staining and comparing counts derived from CDDO-Me-treated N2a cells upon SC16, CVS-11 or CTN infection, with virus-infected N2a cells treated with DMSO serving as a control. Columns and solid circles represent the relative inhibition rate and cell viability, respectively. **G** Culture supernatants were sampled, and the viral titers of the culture supernatants were measured. Comparisons of virus-infected cells treated with each agent at each concentration to the virus-infected, DMSO-treated control group are shown. The data shown represent the mean and standard deviation from two independent experiments that were performed in triplicate. The mean and standard deviation are shown. Statistical analysis was carried out using one-way ANOVA with Dunnett's post hoc test (*p < 0.05, **p < 0.01, ***p < 0.001″)

**Fig. 3:**
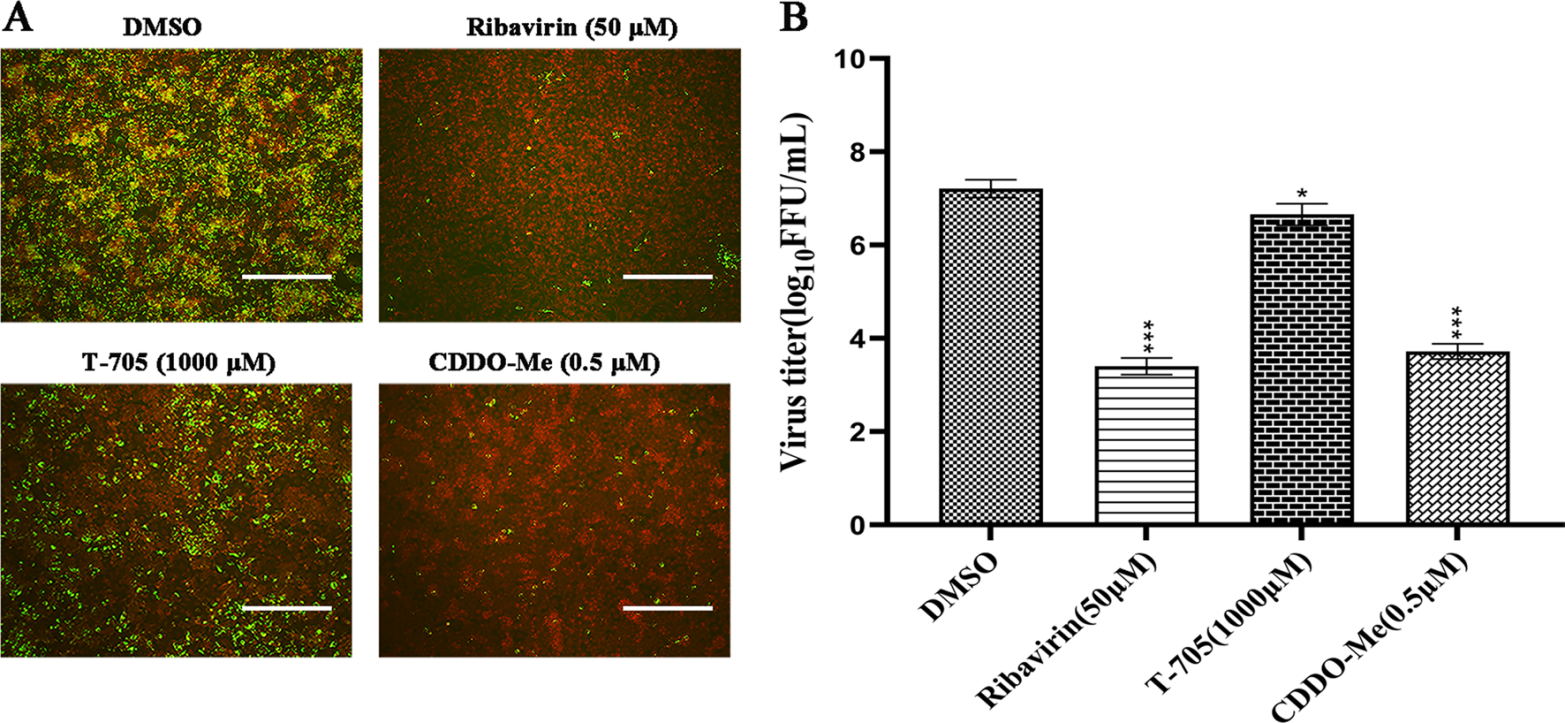
Confirmation of the inhibitory effects of CDDO-Me, ribavirin, and T-705. **A** N2a cells were infected with CVS-11 at an MOI of 10 and treated with different doses of CDDO-Me (0.5 μM), ribavirin (50 μM), or T-705 (1000 μM) for 24 h. Virus-infected cells that were treated with DMSO served as a control in the experiment. The culture supernatants were harvested, and the viral particles were detected by a direct fluorescent antibody (DFA) assay; apple-green fluorescence represents fluorescein isothiocyanate (FITC)-labeled RABV, and the scale bar represents 100 μm. **B** Culture supernatants were sampled, and the viral titers of the culture supernatants were measured. The data shown represent the mean and standard deviation from two independent experiments that were performed in triplicate. The mean and standard deviation are shown. Statistical analysis was carried out using one-way ANOVA with Dunnett's post hoc test (*p < 0.05, **p < 0.01, ***p < 0.001″)

To assess the anti-RABV effects of CDDO-Me in N2a cells infected with different RABV strains (SC16, CVS-11 or CTN), the relative inhibition rates were determined. As shown in Fig. 2 A-F, CDDO-Me significantly inhibited the growth of the SC16, CVS-11 and CTN viruses. The relative inhibition rates ranged from approximately 80% to 95% when CDDO-Me was used at working concentrations of up to 0.5 μM. Importantly, the effects of CDDO-Me against SC16, CVS-11 or CTN infection in N2a cells decreased in a dose-dependent manner. Furthermore, the viral titers of the culture supernatants from N2a cells treated with CDDO-Me and infected with SC16, CVS-11 or CTN were significantly decreased compared with those from N2a cells infected with RABV, suggesting that the replication of RABV strains of differing virulence was severely impaired by CDDO-Me at working concentrations of up to 0.5 μM in N2a cells (Fig. 2G). Since ribavirin and T-705 have demonstrated inhibitory effects against RABV at 50 μM and 1000 μM, respectively, in vitro [6, 14, 46], we examined the inhibitory effects of ribavirin, T-705 or CDDO-Me in N2a cells infected with CVS-11. As shown in Fig. 3A, B, CDDO-Me showed antiviral activity similar to that of ribavirin or T-705 at lower doses in N2a cells. Taken together, the above results show that CDDO-Me significantly inhibited the infection of RABV strains of differing virulence with an efficacy similar to that of ribavirin and significantly better than that of T-705 at lower doses.

### CDDO-Me induced Nrf2-associated pathway activation upon RABV infection in N2a cells

**Fig. 4:**
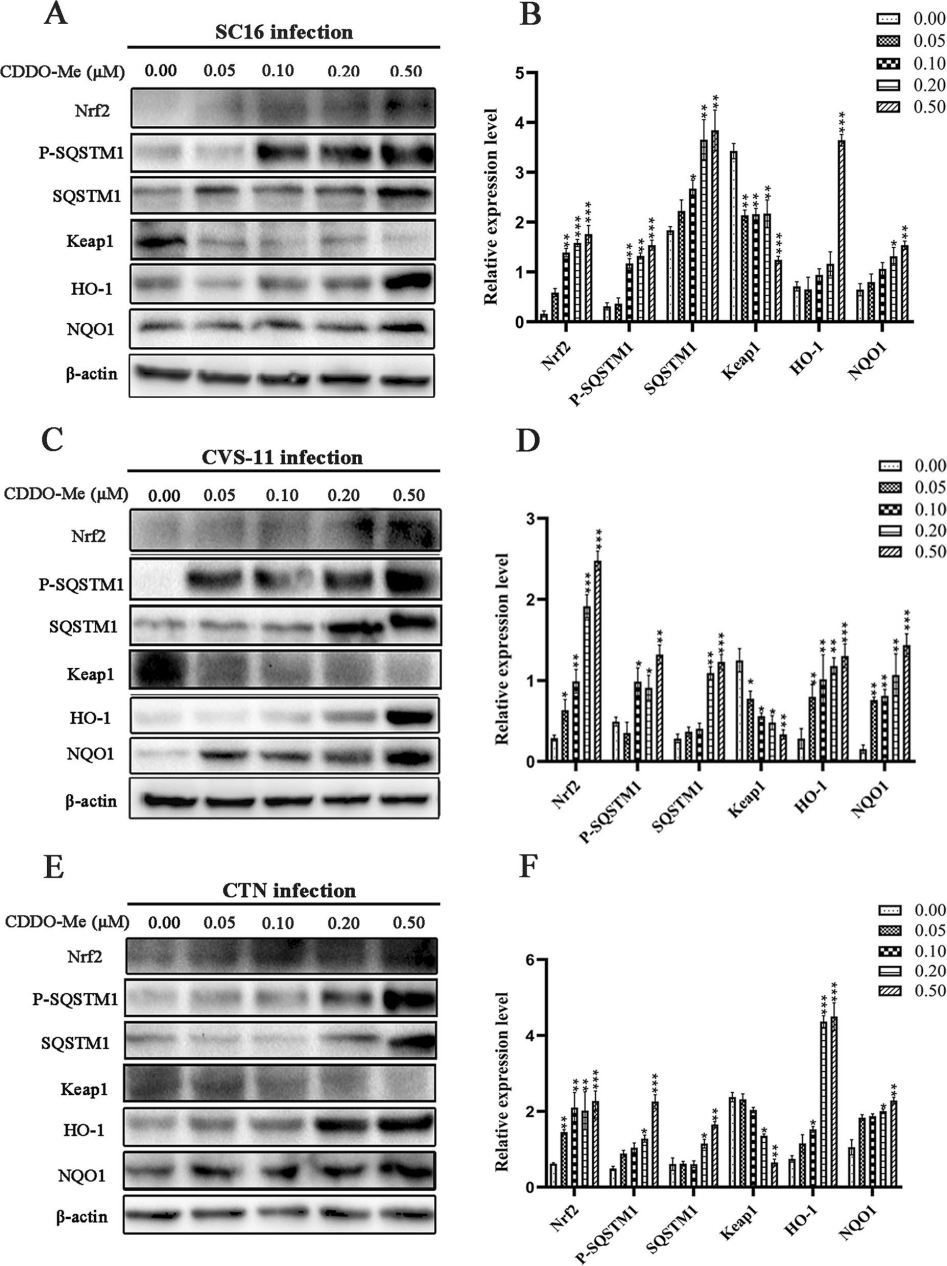
Activation of the Nrf2-associated signaling pathway by CDDO-Me at different concentrations upon infection with different RABV strains in N2a cells**.** N2a cells were infected with SC16 **A**, CVS-11 **C** or CTN **E** at an MOI of 10 and treated with different doses of CDDO-Me (0.05 μM, 0.1 μM, 0.2 μM or 0.5 μM) diluted in culture medium for 24 h in triplicate. Virus-infected cells that were treated with DMSO served as a control. Then, the cells were lysed, and the expression levels of Nrf2, phospho-SQSTM1, SQSTM1, Keap1, HO-1, and NQO1 were examined by western blotting. Data are representative of at least three independent experiments. **B**, **D**, **F**) The results of quantitative analysis of the relative signal densities of Nrf2, phospho-SQSTM1, SQSTM1, Keap1, HO-1, and NQO1 after normalization to the β-actin signal density at the indicated times are shown on the right. Each test was performed in triplicate. Graphical data denote the mean ± SD. Statistical significance was assessed using one-way ANOVA. *p < 0.05, **p < 0.01, ***p < 0.001

Previous studies demonstrated that CDDO-Me and CDDO, two Nrf2 activators in clinical trials, inhibited SARS-CoV-2 replication and the SARS-CoV-2 3C-like protease [47]. Meanwhile, the induction of Nrf2 by another activator could also limit the host inflammatory response and the replication of SARS-CoV-2 [48]. We assessed the interaction of CDDO-Me with the Nrf2-mediated signaling pathway by treating RABV-infected N2a cells with CDDO-Me, which inhibited RABV. First, we examined the levels of members of the Nrf2 pathway in N2a cells infected with the RABV strains SC16, CVS-11 and CTN (which exhibit different levels of virulence) upon CDDO-Me treatment by western blotting (Fig. 4A-F). The expression levels of Nrf2, phospho-SQSTM1, SQSTM1, HO-1 and NQO1 in N2a cells treated with CDDO-Me and infected with SC16, CVS-11 or CTN were increased to different degrees compared with those observed in only infected cells, and CDDO-Me treatment increased their expression in a dose-dependent manner. The lowest levels were observed in the control group. Simultaneously, CDDO-Me treatment rapidly induced the degradation of Keap1 in N2a cells upon SC16, CVS-11 or CTN infection. These results show that the activation of Nrf2 and phosphorylation of SQSTM1 following CDDO-Me treatment in cells infected with RABV strains of differing virulence was correlated with the degradation of Keap1.

### CDDO-Me inhibited RABV infection via the Nrf2-dependent signaling pathway in N2a cells

**Fig. 5:**
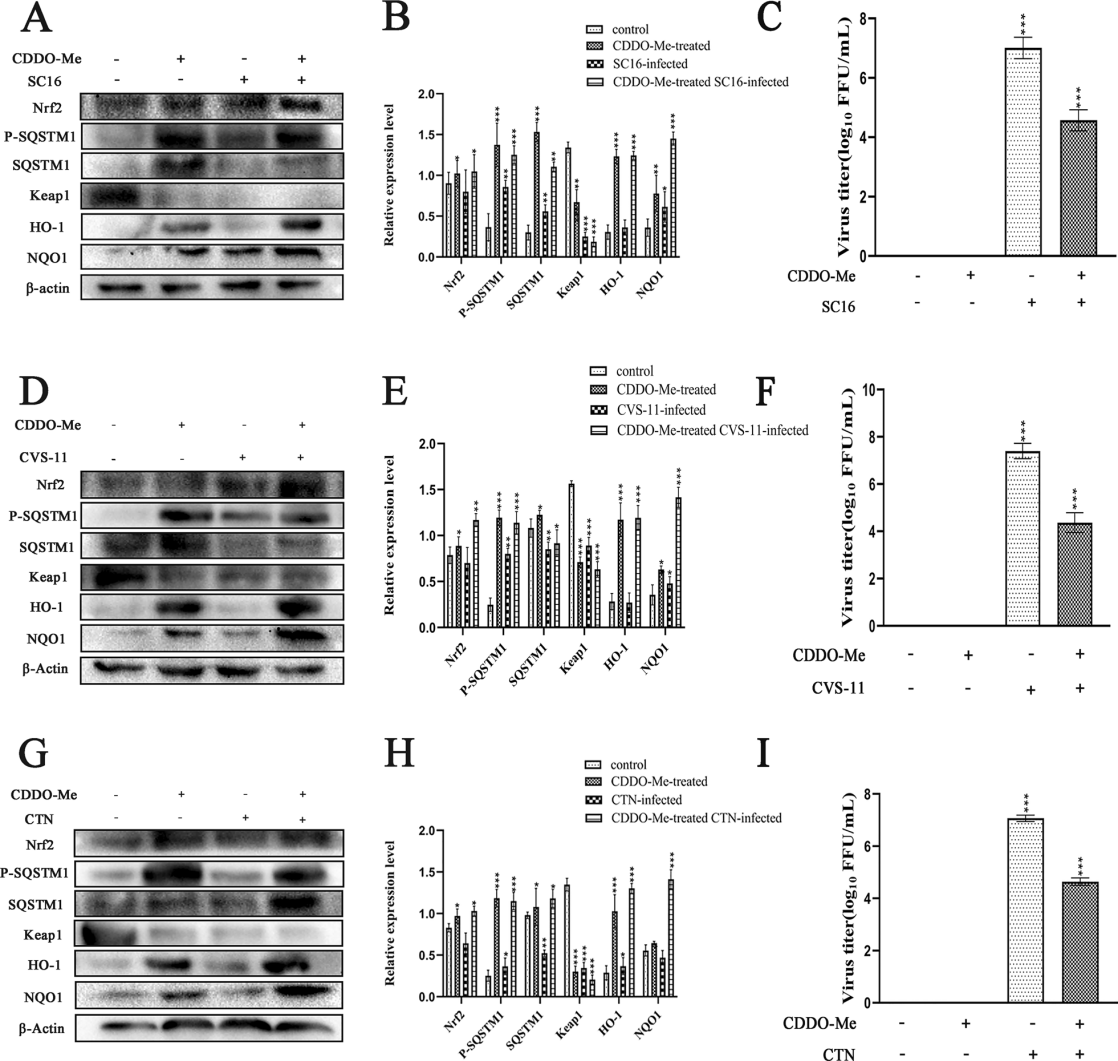
CDDO-Me inhibited different RABV strains via the Nrf2-associated signaling pathway in N2a cells. Nrf2 pathway-associated protein levels were analyzed by western blotting after the infection of N2a cells with SC16 **A**, CVS **D** or CTN **G** at an MOI of 10 and treatment with CDDO-Me (0.5 μM) for 24 h. Virus-infected cells that were treated with DMSO served as a control. Data are representative of at least three independent experiments. **B**, **E**, **H** The results of quantitative analyses of the relative signal densities of Nrf2, phospho-SQSTM1, SQSTM1, Keap1, HO-1, and NQO1 after normalization to the β-actin signal density at the indicated times are shown on the right. **C**, **F**, **I** Culture supernatants were sampled, and the viral titers of the culture supernatants were measured. The data shown represent the mean and standard deviation from two independent experiments that were performed in triplicate. The mean and standard deviation are shown. Statistical analysis was carried out using one-way ANOVA with Dunnett's post hoc test (*p < 0.05, **p < 0.01, ***p < 0.001)

To evaluate whether CDDO-Me inhibits RABV infection via Nrf2 pathway activation, cells were treated with CDDO-Me and infected with SC16; these cells showed increases in Nrf2, phospho-SQSTM1, SQSTM1, HO-1 and NQO1 expression to varying degrees, which were correlated with the degradation of Keap1 (Fig. 5A, B, D, E, G, H). Simultaneously, these inductive effects were also observed in N2a cells treated with CDDO-Me and infected with CVS-11 or CTN. Importantly, HO-1 and NQO1 expression levels were significantly increased in N2a cells treated with CDDO-Me and infected with RABV strains of differing virulence (*P* < 0.01). As shown in Fig. 5C, F, I, N2a cells treated with CDDO-Me at a concentration of 0.5 μM and infected with SC16, CVS-11 or CTN exhibited significantly decreased growth of the SC16, CVS-11 and CTN viruses (*P* < 0.01). These results show that CDDO-Me inhibited the replication of RABV strains of differing virulence via Nrf2 pathway activation.

**Fig. 6:**
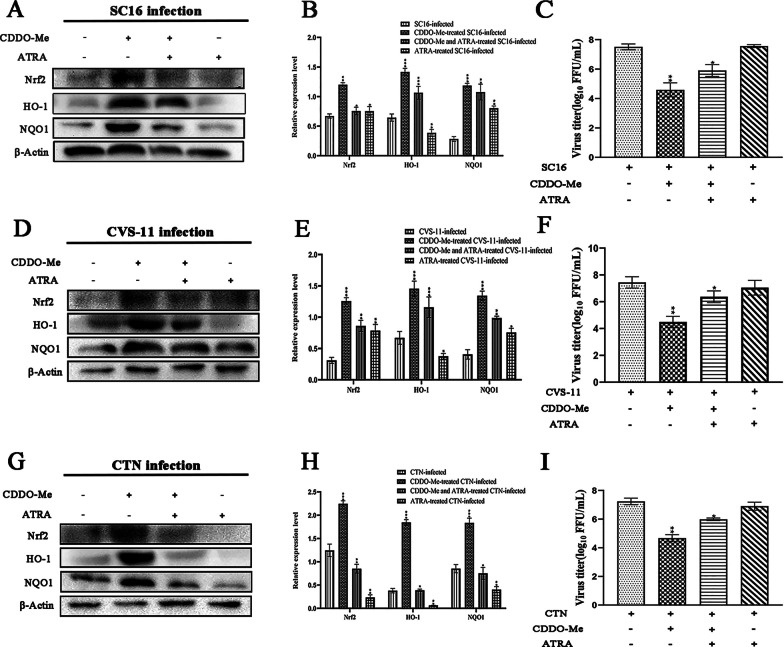
CDDO-Me inhibited different RABV strains via the Nrf2-dependent signaling pathway in N2a cells**.** N2a cells were pretreated with ATRA (10 µM) for 6 h, infected with SC16 **A**, CVS-11 **D** or CTN **G** at an MOI of 10 and treated with CDDO-Me for 24 h. Virus-infected cells that were treated with DMSO served as a control. Nrf2, HO-1 and NQO1 levels were examined by western blotting. Data are representative of at least three independent experiments. **B**, **E**, **H** The results of quantitative analyses of the relative signal densities of Nrf2, HO-1 and NQO1 after normalization to the β-actin signal density at the indicated times are shown on the right. Each test was performed in triplicate. **C**, **F**, **I** Culture supernatants were sampled, and the viral titers of the culture supernatants were measured. Graphical data denote the mean ± SD. Statistical significance was assessed using one-way ANOVA. *p < 0.05, **p < 0.01, ***p < 0.001

Further confirming that CDDO-Me induced Nrf2 pathway activation in a Nrf2-dependent manner, N2a cells that had been pretreated with the Nrf2 inhibitor ATRA before CDDO-Me treatment and SC16 infection showed significantly decreased Nrf2, HO-1 and NQO1 expression (*P* < 0.01). These inhibitory effects were also observed upon infection with CVS or CTN (Fig. 6A, B, D, E, G, H). Moreover, to determine whether CDDO-Me inhibited RABV infection by activating Nrf2, the relative inhibition rates in each group were also detected. As shown in Fig. 6C, F, I, CDDO-Me significantly inhibited the viral growth of the SC16, CVS-11 and CTN strains (*P* < 0.01). Meanwhile, the inhibitory effect of CDDO-Me was diminished when cells were pretreated with ATRA. These data demonstrate that CDDO-Me inhibited RABV infection via the Nrf2-dependent signaling pathway in N2a cells.

## Discussion

Several agents, such as amantadine, interferon alpha, PGG, TMR-001, ribavirin and the two related compounds EICAR and EICNR [6], have been shown to have antiviral activity against RABV in vitro, but no approved RABV-specific antiviral drugs that inhibit RABV infection in clinical cases of RABV after symptom onset are available. Therefore, the need to identify more effective drugs to reduce rabies fatalities is urgent.

CDDO-Me is an FDA-approved compound that has long been known to have anti-inflammatory and exceptional cytoprotective activity [49]. Furthermore, CDDO-Me has been researched in the context of inflammatory disorders, neurodegenerative diseases, lymphoid malignancies, melanoma, chronic kidney disease, diabetes and many other diseases [17, 50]. Due to the known toxicity and inhibitory effect of CDDO-Me in N2a cells, CDDO (a CDDO-Me analog) was also evaluated. In the present study, our data demonstrated that the CC_50_ values of CDDO-Me and CDDO were 0.78 μM and 0.71 μM, respectively, with no apparent cytotoxicity observed at working concentrations of up to 0.5 μΜ in N2a cells. These results are similar to data from Namrata et al., who reported dose-dependent toxicity in which higher concentrations of 0.75 Μm–2 μM were extremely cytotoxic [50]. Qi Sun et al. tested the anti-SARS-CoV-2 activity of CDDO and CDDO-Me and found that CDDO and CDDO-Me could inhibit SARS-CoV-2 replication in Vero cells with EC_50_ values of 0.29 μM (SI = 23.9) and 0.43 μM (SI = 56.6), respectively, and CDDO and CDDO-Me also inhibited SARS-CoV-2 viral replication in human Calu-3 cells at 48 hpi with EC_50_ values of 0.20 μM (SI = 5.8) and 0.42 μM (SI = 28.2), respectively [47]. Here, the anti-RABV activities of CDDO and CDDO-Me were initially examined in N2a cells. To validate the antiviral effects of CDDO and CDDO-Me, the two reference compounds ribavirin and T-705, which have shown inhibitory effects against RABV in vitro, were used for comparisons of efficacy and toxicity [6, 14, 46]. CDDO and CDDO-Me demonstrated high anti-RABV activity similar to that of ribavirin or T-705 in N2a cells. CDDO-Me is more potent than CDDO in terms of its anticancer and cancer-preventive activities and ability to activate the Keap1/Nrf2/ARE pathway, which is involved in oxidative stress [14, 24], and the anti-SARS-CoV-2 activity of CDDO-Me has a greater inhibitory effect on SARS-CoV-2 replication than that of CDDO [47]. Meanwhile, CDDO-Me was observed to have a greater anti-RABV effect in our study; thus, CDDO-Me was chosen to study the related pathways.

CDDO-Me, a Nrf2 activator, has shown great potency against neurological diseases involving oxidative stress and inflammation via Nrf2 activation [50, 51]. It has been recognized for over a decade that oxidative stress is a feature of many viral infections [52], and several studies have reported that oxidative stress plays a role in RABV infection. CVS infection-induced axonal swelling and degeneration and impaired axonal growth were found to be associated with oxidative stress in cultured adult rodent dorsal root ganglion (DRG) neurons [53]. The interaction of a RABV phosphoprotein with mitochondrial complex I induced mitochondrial dysfunction and oxidative stress, which induced neuronal injury [54, 55]. Takagi T and Takahiko I et al. demonstrated that CDDO-Me treatment could improve neurological symptoms, reduce intracranial hemorrhage and ameliorate BBB disruption in cerebral ischemia–reperfusion injury due to the activation of Nrf2 and its target genes in neurons and astrocytes [56, 57]. Studies have shown that Nrf2 disrupts oxidative stress-mediated cell death by scavenging cellular reactive oxygen species (ROS) and restoring redox balance [58], and Nrf2 was suggested to be a pivotal host factor involved in protection against virus-induced cell death that is essential to protect the antioxidant system from oxidative injury and inflammation [29, 31, 32, 34, 59]. Thus, we further explored whether RABV-induced oxidative stress and injury could be improved by the inhibition of viral replication due to CDDO-Me treatment and whether the mechanism was associated with Nrf2 activation in RABV-infected N2a cells. We evaluated the role of Nrf2 in CDDO-Me-treated N2a cells upon infection with different RABV strains and found that the expression levels of Nrf2, phospho-SQSTM1, SQSTM1, HO-1 and NQO1 in N2a cells increased to varying degrees in a dose-dependent manner due to CDDO-Me treatment upon infection with SC16, CVS-11 or CTN. Notably, the levels of HO-1 and NQO1 increased markedly in CDDO-Me-treated N2a cells upon infection with RABV strains of differing virulence. Ana I. Rojo and Robert et al. suggested that Nrf2 is a critical element in survival and death decisions when neurons are exposed to an oxidant environment and proposed the use of Nrf2 agonists as immune therapies for cancer and infection [60, 61]. In vitro and in vivo studies have shown the crucial roles of Nrf2 in neuroprotection and protection against Parkinson’s disease [62, 63]. Moreover, the myelin-preserving, neuroprotective, anti-inflammatory, antiapoptotic and antioxidative stress effects of CDDO-Me treatment were found to be mediated by the regulation of HO-1 and NQO1 in retinal ganglion cells [17]. Castro et al. also demonstrated that treatment with the Nrf2 inducer butylated hydroxyanisole (BHA) could suppress RSV disease phenotypes and enhance viral clearance in murine lungs [59]. Audrey et al. showed that MARV could hijack the Nrf2-antioxidant defense system and that the viral protein VP24 of MARV could bind Keap1 via its Kelch domain, liberating Nrf2 from Keap1 to control and activate the Nrf2 pathway during MARV infection [37]. The Nrf2 activator CDDO-Me has been suggested as an anti-COVID-19 agent that can reduce inflammation, inhibit viral replication, and facilitate cytoprotection and tissue repair [47]. This might explain why Nrf2, HO-1 and NQO1 activation in CDDO-Me-treated N2a cells was observed upon RABV infection and why Nrf2 plays a critical role in RABV inhibition.

The Keap1-Nrf2 signaling axis, which is activated by CDDO-Me, is a master regulator of the response to oxidative/electrophilic stress and chemical insult through the coordinated induction of a wide array of cytoprotective genes [14, 24]. Some results have suggested that the induction of some Nrf2 target genes, such as NQO1, requires Nrf2 and/or Nrf2-associated proteins, which were also found to be upregulated in the livers of Keap1-KD mice [64]. The binding of CDDO-Me to Keap1 disrupts critical cysteine residues in Keap1, leading to the release of Nrf2, which induces nuclear translocation, causing the activation of HO-1 and NQO1 [14, 21, 22, 24]. Our data demonstrate that CDDO-Me significantly inhibited the replication of RABV, accompanied by Nrf2 activation and Keap-1 dissociation, which significantly increased the activation of HO-1 and NQO1. Notably, we also found that the increase in SQSTM1 phosphorylation at Ser349 was significantly associated with the degradation of Keap-1. Studies have reported that SQSTM1, especially upon its phosphorylation at Ser349, can compete with Nrf2 for Keap1 binding, and as a result, SQSTM1 sequesters Keap1 into the autophagosome and prevents Keap1-mediated Nrf2 degradation [21–23]. Da Hyun Lee et al. also found that Nrf2-mediated induction of SQSTM1 activated the noncanonical Keap1–Nrf2 pathway and protected the liver from lipotoxicity in mice [65]. Our data suggest that the function of Keap1 may have been repressed by the phosphorylation of SQSTM1 in CDDO-Me-treated N2a cells upon RABV infection. Notably, we also found that N2a cells pretreated with the Nrf2-specific inhibitor ATRA showed a significant decrease in HO-1 and NQO1 expression and a decrease in the anti-RABV efficacy of CDDO-Me. These inhibitory effects were observed upon infection with RABV strains of differing virulence. As CDDO-Me is a Nrf2 activator, it is thought to be very important in the cell to protect against diseases involving oxidative stress and inflammation via Keap1–Nrf2 pathway activation [51]. Additionally, we found that the levels of p-SQSTM1, along with those of the Nrf2-regulated genes HO-1 and HQO-1, were increased to varying degrees in RABV-infected cells. Recent evidence suggests that Nrf2 acts in viral infection, although the underlying molecular mechanisms remain poorly understood [34, 36–38, 66]. Tetsuya Saito et al. found that SQSTM1 phosphorylated at Ser349 accumulated in tumor regions positive for HCV [29]. Phosphor-STATs 1/3 are known to be involved in ROS signal transduction caused by RSV, and the expression of Nrf2 and downstream ARE-responsive genes was also strongly induced in response to RSV [34]. Chronic infection, a permanent inflammatory process, occurs by an HBV-induced increase in ROS levels triggered by an insufficient immune response. HBV can induce protection against oxidative damage by triggering the expression of Nrf2/ARE-regulated genes. The induction of Nrf2/ARE-regulated genes by HBV protects HBV-positive cells and thereby ensures viral replication [67]. The HIV-1 regulatory protein Tat could also coordinate the induction of ROS production and nuclear Nrf2 accumulation, but it was not sufficient for protection against Tat-induced oxidative stress in multinuclear activation of galactosidase indicator (MAGI) cells [38]. RABV infection is thought to promote ROS generation, causing axonal injury in DRG neurons, mitochondrial dysfunction, and the increased generation of ROS through oxidative stress [53, 55]. Therefore, we speculate that the coordinated induction of ROS production and the induction of Nrf2/ARE-regulated gene expression by RABV protects RABV-positive cells but induces insufficient protection against oxidative damage, leading to viral replication. Meanwhile, Nrf2 activation played a key role in the effects of CDDO-Me treatment in N2a cells, causing RABV inhibition, perhaps by promoting the cytoprotective defense response mediated by CDDO-Me. The increase in HO-1 and NQO1 expression observed in those N2a cells was triggered in the context of the anti-RABV effects of CDDO-Me through activation of the Nrf2 signaling cascade.

In summary, our data demonstrate that CDDO-Me could inhibit RABV infection in a manner dependent on Nrf2 activation in N2a cells, indicating that treatment with this compound serves as a therapeutic strategy for inhibiting RABV and protecting neurons during viral infection.

## Conclusions

CDDO-Me significantly inhibited three RABV strains of differing virulence (SC16, CVS-11 and CTN), and its anti-RABV activity was similar to that of ribavirin or T-705 in N2a cells. The role of the Nrf2 pathway in CDDO-Me-treated N2a cells upon infection with different RABV strains was evaluated, and the expression levels of Nrf2, phospho-SQSTM1, SQSTM1, HO-1 and NQO1 in N2a cells increased to varying degrees in a dose-dependent manner due to CDDO-Me treatment upon infection with SC16, CVS-11 or CTN. Notably, the levels of HO-1 and NQO1 increased markedly. Moreover, CDDO-Me significantly inhibited the replication of RABV, accompanied by Nrf2 activation and Keap-1 dissociation, which significantly increased the activation of HO-1 and NQO1. The activation of SQSTM1 phosphorylation at Ser349 was significantly associated with the degradation of Keap-1 in CDDO-Me-treated N2a cells upon RABV infection. Meanwhile, N2a cells pretreated with the Nrf2-specific inhibitor ATRA showed a significant decrease in HO-1 and NQO1 expression and a decrease in the anti-RABV efficacy of CDDO-Me. These inhibitory effects were observed upon infection with RABV strains of differing virulence. The data obtained here provide direct evidence that CDDO-Me could inhibit RABV infection in a manner dependent on Nrf2 activation in N2a cells. The present study provides a therapeutic strategy for inhibiting RABV and protecting neurons during viral infection.

## Data Availability

The materials described in the manuscript will be made freely available to any scientist wishing to use them.

## Abbreviations

RABV: Rabies virus
CNS: Central nervous system
CDDO-Me: Bardoxolone methyl
Nrf2: Nuclear factor erythroid-derived 2-like 2
SQSTM1: Sequestosome 1
Keap1: Kelch-like ECH-associated protein 1
HO-1: Heme oxygenase 1
NQO1: NAD(P)H:quinone oxidoreductase 1
DFA: Direct fluorescent antibody
WB: Western blot
